# Reliability of Ocular Surface and Biometric Measurements Under Face Mask Exposure: A Narrative Review

**DOI:** 10.3390/jpm16100521

**Published:** 2026-10-08

**Authors:** Guillermo Rodríguez Iranzo, Cristina Peris Martínez

**Affiliations:** 1 Fundación de Oftalmología Médica de la Comunidad Valenciana (FOM), Pío Baroja 12, 46015 Valencia, Spain; 2 Surgery Department-Ophthalmology, University of Valencia, Blasco Ibáñez 15, 46010 Valencia, Spain

**Keywords:** narrative review, preoperative optimization, optical biometry, mask-associated ocular symptoms, swept-source optical coherence tomography, patient stratification, ocular surface disease

## Abstract

**Background**: The widespread global adoption of facial respiratory barriers across diverse clinical and public environments has led to a significant increase in specialized consultations regarding unexpected ocular irritation and surface dryness. This clinical presentation, often secondary to mask-associated ocular symptoms, represents a unique challenge for accurate preoperative assessment in cataract surgery. This narrative review provides a comprehensive and critical evaluation of the literature exploring the relationship between mask-induced microenvironmental stress, patient-reported symptoms and objective tear film parameters, focusing specifically on their distinct impacts on preoperative ocular biometry and diagnostic measurement reliability for cataract surgery planning. **Methods**: A screening of published clinical studies and cohorts evaluating questionnaires, non-invasive tear film kinetics, epithelial structural changes and high-resolution automated topography was conducted across major biomedical databases up to April 2026 to evaluate underlying patterns, clinical associations and research gaps. **Results**: The available evidence suggests a distinct and reproducible dissociation between ocular surface symptoms and objective parameters. While improperly fitted superior mask margins may direct continuous, asymmetric warm convective air currents upward over the periorbital region—a mechanism hypothesized to accelerate tear evaporation and contribute to ocular surface discomfort—the evidence regarding biometric technology vulnerability remains constrained to limited sample sizes. Early automated measurements, particularly deeper metrics such as axial length, show good reproducibility under specific mask exposure settings, whereas superficial tear-film-dependent measurements require careful isolation. **Conclusions**: Strategic preoperative pathways must differentiate acute, transient microenvironmental alterations from persistent or progressive pre-existing ocular surface disease. Clinical screening workflows should incorporate cautious terminology and the optimization of mask fit or targeted mask removal should be actively considered during automated data acquisition, particularly for patients with compromised ocular surfaces. This structured approach incorporates targeted ocular surface assessment and management, including optimization of mask fit and tear film therapy when clinically indicated, to guarantee patient comfort and predictable postoperative visual outcomes.

## 1. Introduction

The paradigm of contemporary lens extraction has evolved from a routine anatomical replacement procedure into a highly customized refractive intervention aimed at optimizing postoperative visual quality and patient satisfaction. Patients undergoing phacoemulsification increasingly expect excellent uncorrected vision across multiple distances, minimal residual refractive error and reduced dependence on spectacles. To meet these expectations, clinicians rely on advanced biometric formulas, artificial intelligence-based prediction models and automated diagnostic technologies. Despite substantial advances in intraocular lens (IOL) power calculation, postoperative refractive errors exceeding ±0.5 diopters still occur in approximately 20–40% of eyes, highlighting the need to identify and minimize subtle sources of biometric variability during the preoperative assessment [1,2,3].

The cornea is the main refractive component of the eye, contributing approximately 70% of its total optical power. Because modern biometers estimate corneal power through optical measurements of the anterior corneal surface, the integrity and stability of the ocular surface are essential for obtaining reliable keratometric measurements. The widespread use of face masks in healthcare settings has drawn attention to mask-associated ocular irritations and transient evaporative changes, frequently grouped under the term mas-associated dry eye (MADE), a condition caused by upward airflow from poorly fitted masks that accelerates tear evaporation, destabilizes the tear film and disrupts ocular surface homeostasis [4,5]. However, it is clinically vital to separate these transient, airflow-induced symptoms from true, established dry eye disease, as clear and standardized diagnostic criteria specifically validating MADE as an independent pathology do not exist. These alterations may compromise the reproducibility and accuracy of corneal measurements obtained during preoperative biometry.

Although the American Society of Cataract and Refractive Surgery Cornea Clinical Committee recommends identifying and treating ocular surface disease before biometric evaluation [6], the clinical significance of transient mask-induced ocular surface alterations remains uncertain. Whether airflow-induced dry eye produces temporary measurement variability or clinically relevant changes that influence IOL power calculations has not been fully established. This narrative review critically summarizes the current evidence regarding the effects of mask wear on corneal measurements and cataract surgery planning, identifies existing knowledge gaps and discusses strategies to optimize preoperative ocular surface assessment and improve refractive outcomes.

## 2. Materials and Methods

### 2.1. Search Strategy and Information Sources

A comprehensive narrative literature research was conducted in major medical databases—including PubMed/MEDLINE, Embase and the Cochrane Central Register of Controlled Trials—to investigate the relationship between face mask use, tear film alterations, ocular surface disease and biometric outcomes. The search strategy combined Medical Subject Headings and free-text keywords. Search terms included “face mask”, “respiratory barrier”, “mask-associated dry eye”, “tear film stability”, “ocular surface disease”, and “optical biometry”.

The database search was initially executed up to April 2026. Given the lack of a pre-registered systematic screening protocol, comprehensive tracking of excluded records, or independent dual-reviewer quality scoring, this text is explicitly presented as a narrative review. Consequently, the conclusions drawn are subject to the methodological limitations of non-systematic evidence syntheses, including potential selection bias and the lack of a formal risk-of-bias grading for the compiled publications.

### 2.2. Inclusion and Exclusion Criteria

The review included peer-reviewed studies published in English that investigated the effects of face mask use on the ocular surface or preoperative biometric measurements. Eligible study designs included randomized controlled trials, prospective and retrospective cohort studies and observational investigations. Studies were included if they evaluated validated dry eye questionnaires, tear film parameters, ocular surface integrity or biometric measurements obtained during face mask use.

Animal studies, conference abstracts, narrative reviews, editorials, non-peer-reviewed preprints and studies with insufficient methodological data were excluded.

### 2.3. Data Extraction and Clinical Metrics

Data extraction was performed systematically to evaluate the clinical effects of face mask use on the ocular surface and preoperative biometry. Extracted variables were grouped into three predefined domains:

- Subjective symptom assessment: validated patient-reported outcome measures, including the Ocular Surface Disease Index (OSDI) and the Standard Patient Evaluation of Eye Dryness (SPEED) questionnaire.
- Objective ocular surface and biometric parameters: tear break-up time (TBUT), non-invasive tear break-up time (NITBUT), tear meniscus height (TMH), corneal fluorescein staining and biometric measurements including central corneal thickness, axial length and keratometry.
- Molecular and cellular biomarkers: tear inflammatory markers, matrix metalloproteinase-9 (MMP-9), conjunctival cytology, globet cell density and mucin (MUC5AC) expression, where available.

### 2.4. Synthesis and Critical Evaluation

The compiled evidence was critically synthesized to identify clinical patterns, knowledge gaps and the potential effects of different types of face masks (e.g., surgical masks and N95 respirators) on the ocular surface and preoperative biometry. Due to the heterogeneity of study populations, study designs, exposure durations and outcome measures, a qualitative synthesis was performed. The analysis compared subjective symptoms with objective ocular surface and biometric findings to assess the consistency of the available evidence. Particular attention was given to the effects of upward airflow associated with face mask wear on tear film stability, ocular surface homeostasis and the reliability of biometric measurements.

## 3. Results

### 3.1. Pathophysiological Mechanisms of Microenvironmental Ocular Stress

A critical evaluation of the literature indicates that deterioration of tear film homeostasis under respiratory barrier wear is primarily associated with upward airflow generated when a standard surgical or hygienic facial barrier lacks an adequate superior facial seal. Exhaled breath—characterized by increased relative humidity, temperature and carbon dioxide concentration—is redirected toward the nasal and maxillary skin pathways. This localized airflow continuously traverses the exposed interpalpebral fissure, altering the tear film microenvironment by modifying the water pressure gradient, thereby accelerating aqueous tear evaporation and promoting tear film instability and premature break up [7].

This continuous airflow induces mechanical, thermal and functional changes on the ocular surface. High-resolution infrared thermographic profiling has demonstrated a localized temperature increase in the periocular area during active mask wear. This thermal shift may alter the physicochemical properties and stability of the tear film lipid layer, predisposing it to focal disruption and premature break up. Concurrently, the steady airflow has been associated with altered blink dynamics, including an increased frequency of incomplete blinks and reduced blink efficiency. This mechanical limitation impairs the homogeneous redistribution of meibomian lipids across the ocular surface, thereby exacerbating evaporative stress, ocular surface desiccation and friction-related epithelial damage [8].

### 3.2. Disparities Between Subjective Symptoms and Objective Signs

#### 3.2.1. Subjective Symptom Assessment

The evaluation of subjective symptoms constitutes the primary clinical approach for assessing mask-associated symptoms. The literature consistently indicates that forced upward airflow accelerates tear evaporation, triggering stimulation of corneal sensory nerve endings. Consequently, patients frequently experience substantial ocular discomfort that may not correlate with objective structural alterations in the ocular surface. This sensory disturbance manifests through a characteristic spectrum of symptoms, including persistent foreign body sensation, grittiness, ocular burning, dry eye awareness, photophobia, eye redness and fluctuating or transient visual blurring [4,5].

To quantify this functional distress, clinical trials have successfully implemented standardized, validated symptom-intensity metrics. The most prominent tools include the OSDI, which evaluates symptoms and environmental triggers over a past timeframe and the SPEED questionnaire, which assesses the frequency and severity of dry eye symptoms [9,10,11]. Longitudinal and cross-sectional studies consistently demonstrate a significant increase in symptom scores following continuous mask wear, particularly after approximately 30 min of uninterrupted use.

A critical review of the data presented in Table 1 reveals considerable methodological heterogeneity in the assessment of this symptomatic spectrum. Large-scale population-based studies, such as those conducted by Boccardo (*n* = 3605) [11] and Fan et al. (*n* = 6925) [12], consistently demonstrate that dry eye symptoms are significantly associated with mask use, identifying female sex and pre-existing dry eye disease as independent risk factors. Conversely, studies evaluating specific populations, including medical students investigated by Al-Dolat et al. (*n* = 1219) [13] and university cohorts assessed by Giannaccare et al. (*n* = 20) [14], reported significant deterioration in OSDI scores despite relatively shorter exposure times, suggesting that concomitant factors, particularly prolonged digital screen exposure, may contribute to or potentiate mask-associated evaporative stress. Similarly, Alanazi et al. [15] reported that 1 h of continuous mask wear significantly worsened SPEED scores, indicating an increase in subjective ocular symptoms among healthy individuals (*n* = 54).

Furthermore, occupational studies by Dag et al. [16] and Erogul et al. [17] demonstrated a significant association between daily mask-wearing during working hours and the severity of subjective ocular discomfort among healthcare professionals. This group represents a clinically relevant population, as prolonged mandatory mask use may increase cumulative ocular surface stress. Corneal topographies or surface validation trials by Shalaby et al. [18] introduced an important comparison by analyzing mask designs, documenting that standard surgical masks induced a more severe subjective OSDI shift than tightly fitted N95 respirators. These findings suggest that inadequate sealing of the superior mask edge facilitates greater upward airflow toward the ocular surface, thereby increasing tear film evaporation and ocular discomfort.

#### 3.2.2. Objective Sign Assessment

To fully characterize the clinical impact of mask wear, the literature has focused on objective parameters that capture changes in the stability, volume and structure of the precorneal tear film. The continuous convective airflow directed toward the periocular area may create localized microenvironmental changes that accelerate tear evaporation. Objective testing protocols are designed to assess whether this transient evaporative stress produces persistent ocular surface alterations or introduces clinically relevant changes in diagnostic measurements during preoperative screening.

As summarized in Table 2, the objective sign profile demonstrates a unique pattern of localized tear film disruption while overall macrostructural ocular architecture remains largely preserved. A critical analysis of tear film kinetics shows that Shalaby et al. [18] and Azzam et al. [19] both confirmed significant post-wear reductions in TBUT, consistent with increased tear film instability and lipid-layer disruption. However, Azzam et al. observed that N95 models caused a more severe objective reduction in TBUT than surgical masks. It has been proposed as a hypothesis that improperly fitted N95 masks might generate a localized, higher-pressure air jet producing deeper lipid-layer desiccation if misaligned. Nevertheless, this remains inferential and represents an unproven statement because direct micro-airflow imaging was not conducted [19].

Further mapping of non-invasive dynamics by Alanazi et al. [15] and Esen Baris et al. [9] tracked immediate, significant reductions in NITBUT during short periods of mask wear, supporting the role of convective airflow in promoting tear film instability without the need for invasive fluorescein manipulation. Similarly, Giannaccare et al. [14] documented a significant reduction in TMH after an 8-h testing protocol, suggesting that prolonged exposure may progressively reduce tear volume.

However, these changes in tear film dynamics do not appear to be translated into cellular or structural alterations. D’Souza et al. [20] offered valuable biological insights by performing comprehensive lacrimal protein assays. While they found no structural modifications in TBUT or Schirmer parameters in their controlled cohort (*n* = 17), they reported distinct subclinical changes, including increased tear concentrations of leukocytes and natural killer cells, together with downregulation of B-cell expression. This profile may indicate a localized ocular surface immune response [20].

Similarly, Marta et al. [21] analyzed historical cohorts (*n* = 274) across pre- and post-pandemic timelines, documenting long-term changes in blink frequency, TMH and tear osmolarity during periods of mandatory mask wear, while noting that basic lipid layer thickness and Schirmer scores remained stable or showed paradoxical improvement, potentially reflecting compensatory reflex lacrimal gland activity [21].

Burgos-Blasco et al. [22] evaluated macro-optical variables using high-resolution Scheimpflug tomography, reporting that mask-associated ocular surface changes did not significantly alter flat, steep or mean keratometric parameters. These findings suggest that, despite measurable changes in tear film dynamics and selected ocular surface biomarkers, the macrostructural and keratometric parameters relevant to automated diagnostic calculations remain relatively stable.

Finally, in a recent study evaluating the impact of face masks on cataract surgery planning [23], 58 eyes of 29 patients were analyzed using the optical biometer IOLMaster 700. The methodological design compared repeated intra-subject measurements taken after wearing a surgical mask for at least 30 min against measurements obtained after a 10-min mask-free period under controlled environmental conditions. The authors reported no statistically significant differences in central corneal thickness, keratometry, axial length or the final IOL power calculations between both situations. Consequently, a high clinical concordance was established for biometric parameters regardless of mask wear, although individual evaluation of mask-associated ocular surface symptoms is recommended.

### 3.3. Molecular Biomarkers and Subclinical Inflammation

At the cellular level, the hyperosmolar environment induced by mask-associated tear evaporation may trigger subclinical inflammatory pathways. Continuous cellular desiccation may stimulate the corneal and conjunctival epithelia to activate mitogen-activated protein kinase pathways, thereby promoting the release of pro-inflammatory cytokines [24]. Biochemical analysis of tear samples from symptomatic mask wearers has revealed increased expression of MMP-9, a reliable clinical biomarker of extracellular matrix degradation, ocular surface stress and epithelial barrier disruption [25].

Furthermore, prolonged exposure to convective currents may compromise superficial epithelial protection. Impression cytology has demonstrated a notable downregulation of gel-forming mucins, particularly MUC5AC, which is secreted by conjunctival globet cells. This localized mucin deficiency may reduce the hydrophilicity of the corneal surface, potentially creating a more vulnerable epithelial interface [25]. While these molecular markers indicate subclinical inflammation and epithelial stress, the claim that MMP-9 elevation or reduced MUC5AC translates directly into delayed postoperative epithelial healing or poorer visual outcomes with multifocal or toric intraocular lenses (IOLs) is overextended. These clinical consequences must be framed strictly as prospective hypotheses, given that direct clinical evidence correlating these specific biomarkers with poor post-surgical outcomes in mask wearers is currently unavailable [25].

### 3.4. Current Knowledge Gaps and Structural Problems

Despite extensive documentation, several critical gaps persist in the current literature:

- Lack of standardized diagnostic thresholds: Most published studies rely on generic dry eye criteria to diagnose these complaints [26,27]. There remains a distinct lack of standardized, specific diagnostic thresholds capable of differentiating transient mask-related microenvironmental changes from chronic, progressive ocular surface disease.
- Undefined exposure thresholds: The exact duration and intensity of exposure to evaporative stress required to induce persistent structural or cellular changes remain unclear [25]. Longitudinal studies evaluating long-term cellular and structural modifications are currently lacking in the literature.
- Vulnerability to confounding factors and lack of quality assessments: A major limitation of the existing literature is that a high proportion of the studies summarized in Table 1 rely heavily on questionnaire-based or cross-sectional architectures. These designs are highly susceptible to critical confounding variables, including digital screen exposure time, environmental humidity, baseline dry eye severity, precise mask fit variations and actual duration of continuous mask use. Furthermore, no formal risk-of-bias or structured evidence-quality assessment has been executed across these aggregated datasets, limiting generalized conclusions.
- Inconsistent device-specific artifact correction: while advanced swept-source optical coherence tomography (SS-OCT) hardware appears to demonstrate greater resistance to surface irregularities [28], older diagnostic setups—such as standard Placido-disk topographers or older partial coherence interferometers—are still widely used globally. The relative susceptibility of these different diagnostic modalities to mask-induced airflow and tear film instability has not yet been fully quantified.

## 4. Discussion

The clinical evidence integrated within this review highlights a fundamental pathophysiological distinction: superficial tear film variations do not automatically translate into deep anatomical shifts. To optimize preoperative cataract planning, workflows must clearly separate clinical observations into three distinct categories: immediate ocular surface microenvironmental alterations, technology-specific measurement reliability and the subsequent clinical implications for IOL calculation [23,29]. While superficial tear-film-dependent parameters, such as keratometry, remain inherently vulnerable to acute airflow currents, deeper structural parameters, like axial length, maintain high reproducibility even under continuous mask exposure.

Furthermore, the current literature regarding biometric safety under respiratory barrier exposure depends on restricted sample sizes, primarily constrained to limited studies. The field relies mainly on one small Pentacam study involving 35 healthy subjects [22] and one IOLMaster 700 trial containing 58 eyes of 29 cataract patients [23]. These limited data are statistically insufficient to conclude universally that all modern biometric technologies are completely unaffected by mask wear or that clinical workflows require zero adaptation. Therefore, the prior recommendation that mask removal is generally unnecessary must be softened; it does not establish safety for all mask variants, high-risk irregular corneas or distinct diagnostic biometers.

Rather than proposing definitive clinical mandates, patient stratification must guide clinical decision-making. A clear distinction must be established between a healthy individual presenting with temporary, minor mask-associated symptoms and a cataract candidate with significant pre-existing evaporative dry eye or meibomian gland dysfunction. While the former group exhibits transient changes, the latter group is highly relevant when discussing the reliability of preoperative keratometry and premium (toric or multifocal) IOL planning [29]. In patients with pre-existing ocular surface disease, any additional airflow-induced tear destabilization can exacerbate baseline corneal measurement noise, increasing the risk of refractive surprises.

To improve readability and remain aligned with verified findings, speculative discussions regarding personalized medicine models, automated machine learning biometric filtering and distant future molecular screening technologies have been curtailed, as they extend beyond what is supported by the current literature [23]. Instead, clinical decision-making should integrate the relative reproducibility of deeper biometric metrics with an active optimization of the ocular surface before data acquisition [6].

## 5. Conclusions

This narrative review demonstrates that while deeper automated structural metrics like axial length show stability in early, small-scale testing, superficial tear-film-dependent parameters such as keratometry demand a more cautious clinical approach. The current evidence remains limited by small sample sizes and potential environmental confounders. Ophthalmic clinicians should not assume complete biometric immunity to mask airflow. Instead, clinical workflows should incorporate targeted patient stratification, careful pre-examination mask adjustment, and consideration of temporary mask removal during automated keratometry or topography acquisition—particularly in individuals with pre-existing ocular surface disease—to ensure optimal data accuracy and predictable postoperative visual outcomes.

## Figures and Tables

**Table 1:** Comprehensive synthesis of the primary literature evaluating subjective symptomatology associated with face mask wear.

Authors & Study Type	Study Population	Clinical Tests	Primary Ocular Surface Observations
Boccardo [11] Cross-sectional, descriptive, observational	General population (*n* = 3605)	Online questionnaire	2447 individuals experienced dry eye symptoms; among these, 658 (26.9%) exhibited a marked increase in symptomatology directly associated with face mask use.
Fan et al. [12] Cross-sectional, descriptive, observational	General population (*n* = 6925)	Online questionnaire	419 participants experienced new-onset dry eye symptoms, and 128 reported an exacerbation of pre-existing symptoms due to mask wear.
Al-Dolat et al. [13] Cross-sectional, descriptive, observational	Medical students (*n* = 1219)	Online questionnaire and OSDI scale	546 individuals (44.8%) wore masks for at least 3 h per day and 874 (71.7%) met the clinical criteria for dry eye according to OSDI scoring thresholds.
Alanazi et al. [15] Prospective longitudinal, analytical, observational	Healthy individuals (*n* = 54) and controls without mask (*n* = 50)	SPEED questionnaire	Mask use for a 1-h interval significantly reduced comfort scores on the scale, representing a distinct increase in subjective symptoms.
Giannaccare et al. [14] Cross-sectional, analytical, observational	University students (*n* = 20)	Standardized questionnaire and OSDI scale	Active face mask wear caused a significant degradation in OSDI scores, which worsened from a baseline of 12.9 ± 12.6 to 19.4 ± 12.0.
Dag et al. [16] Cross-sectional, analytical, observational	Healthcare professionals (*n* = 333)	Online questionnaire	The documented prevalence of mask-associated symptoms was 70%. Primary risk factors included advanced age and the baseline presence of at least one dry eye symptom without a mask.
Erogul et al. [17] Cross-sectional, analytical, observational	Healthcare professionals (*n* = 396)	Online questionnaire	Identified a statistically significant correlation between total mask wear duration and ocular pain, with conjunctival redness being the most prevalent sign.
Shalaby et al. [18] Prospective longitudinal, analytical, observational	Healthy individuals (*n* = 200:100 surgical mask and 100 N95)	OSDI scale	Significant escalation of OSDI scores manifest in both study groups; this clinical variation was more pronounced in users wearing surgical masks compared to N95 models.

**Table 2:** Comprehensive synthesis of the primary literature measuring objective ocular surface signs associated with face mask wear.

Authors & Study Type	Study Population	Clinical Tests	Primary Ocular Surface Observations
Shalaby et al. [18] Prospective longitudinal, analytical, observational	Healthy individuals (*n* = 200:100 surgical mask and 100 N95)	TBUT, Schirmer I and corneal fluorescein staining	All baseline tear film parameters deteriorated significantly after 1 h of continuous mask wear in both groups; surgical masks induced worse outcomes than N95 models.
Azzam et al. [19] Cross-sectional, analytical, observational	Healthcare professionals (*n* = 60:30 surgical mask and 30 N95)	TBUT, corneal/conjunctival staining and meibography	Both barrier types triggered ocular surface dryness according to TBUT values alongside meibomian gland loss; N95 models induced greater dry eye changes via TBUT.
Alanazi et al. [15] Prospective longitudinal, analytical, observational	Healthy individuals (*n* = 54) and controls without mask (*n* = 50)	NITBUT, phenol red thread test and tear ferning test	Statistically significant differences were isolated exclusively in NITBUT values captured before and 1 h after mask wear; remaining test protocols showed no differences.
Esen Baris et al. [9] Cross-sectional, analytical, observational	Healthcare professionals (*n* = 33)	NITBUT and manual TBUT calculations	Mean TBUT values significantly dropped from a baseline morning score of 9.3 ± 1.0 s (range 3–16 s) at 8:00 AM down to 8.3 ± 1.5 s at 5:00 PM (*p* = 0.01) following full-day wear.
Giannaccare et al. [14] cross-sectional, analytical, observational	Healthy university students (*n* = 20)	NITBUT, TMH, ocular redness, and meibography	Following an 8-h continuous mask wear protocol, TMH exhibited a statistically significant reduction; the remaining surface variables remained statistically unchanged.
D’Souza et al. [20] Prospective longitudinal, analytical, observational	Healthy individuals (resident ophthalmologists, *n* = 17)	Schirmer I, TBUT, tear interferometry, OSI, ocular staining, and tear protein assays	No significant changes manifest in structural TBUT, Schirmer I, or OSI after mask use; tear assays revealed elevated leukocytes and natural killer cells alongside lower B cells.
Marta et al. [21] Retrospective longitudinal, analytical, observational	General population (pre- vs. post-pandemic cohorts, *n* = 274)	NITBUT, lipid layer thickness, blink rate, Schirmer, TMH, tear osmolarity, meibography	During active mask periods, blink frequency, TMH and tear osmolarity significantly worsened; conversely, lipid thickness and Schirmer scores improved while NITBUT values were similar.
Burgos Blasco et al. [22] Prospective with repeated measures	35 eyes of 35 healthy individuals with no prior ocular diseases (mean age: 33.5 years; 74% female).	Corneal topography (Pentacam) performed in two phases: two measurements with a face mask (mean wear time: 3.8 h) and two measurements after a 10-min mask-free period. Evaluated parameters included K1, K2, Km, and Kmax.	No statistically significant differences were found in K1, K2, Km, or Kmax between mask and no-mask measurements. Although intraclass correlation coefficients were excellent across all parameters, they were slightly lower when measurements were taken with the face mask.
Rodríguez Iranzo et al. [23] Prospective with repeated measures	58 eyes of 29 patients scheduled for cataract surgery (mean age: 70.2 years).	Biometric parameters (IOLMaster 700) obtained in two phases: with a surgical face mask and without it (after a 10-min rest).	No statistically significant differences were found in biometric values or IOL power calculations between mask and no-mask measurements. High overall concordance.

## Data Availability

No new data were created or analyzed in this study. Data sharing is not applicable to this article.
